# The Role of Retinal Pigment Epithelial Cells in Regulation of Macrophages/Microglial Cells in Retinal Immunobiology

**DOI:** 10.3389/fimmu.2021.724601

**Published:** 2021-08-13

**Authors:** Andrew W. Taylor, Samuel Hsu, Tat Fong Ng

**Affiliations:** Department of Ophthalmology, Boston University School of Medicine, Boston, MA, United States

**Keywords:** ocular immune privilege, immune tolerance, retinal pigment epithelial cells (RPE), retinal immunobiology, suppressor macrophages

## Abstract

The ocular tissue microenvironment is immune privileged and uses several mechanisms of immunosuppression to prevent the induction of inflammation. Besides being a blood-barrier and source of photoreceptor nutrients, the retinal pigment epithelial cells (RPE) regulate the activity of immune cells within the retina. These mechanisms involve the expression of immunomodulating molecules that make macrophages and microglial cells suppress inflammation and promote immune tolerance. The RPE have an important role in ocular immune privilege to regulate the behavior of immune cells within the retina. Reviewed is the current understanding of how RPE mediate this regulation and the changes seen under pathological conditions.

## Introduction

### Ocular Immune Privilege

The eye is called immune privileged from the original observations of prolonged allograft survival within the anterior chamber even following immunization to alloantigens (1). This now includes immune regulation and immune tolerance to antigens and pathogens within the eye (2–4). The ocular microenvironment is delineated by blood barriers and the lack of direct lymphatic drainage. Within this microenvironment immune cells differentiate into cells that suppress inflammation and promote immune tolerance (5). The mediators of ocular immune regulation and tolerance are soluble, and membrane bound molecules. An important membrane bound molecule is membrane FasL (6, 7). Its expression by cells of the ocular blood-barriers, including the retinal pigment epithelial cells (RPE), mediates a contact dependent induction of apoptosis in monocytes and lymphocytes preventing their accumulation and infiltration. Also, the RPE release extracellular membranes expressing membrane-FasL that also induce apoptosis in macrophages potentially away from the RPE monolayer (8). Many of the soluble mediators of ocular immunosuppression can be found in aqueous humor and in the supernatant of cultured RPE. These mediators include a wide range immunoregulating cytokines, neuropeptides, and soluble ligands. These include Transforming Growth Factor-beta2 (TGF-β2) and alpha-Melanocyte Stimulating Hormone (α-MSH), which are highly conserved and potent regulators of immune cell activity and suppressors of inflammation (9–12). The current picture of ocular immune privilege is a tissue microenvironment that actively manipulates immune cells to promote the health of the visual axis, and to prevent the activation of inflammation. These mechanisms of ocular immune privilege are for most of us highly effective in preventing inflammation, and the mediators of immune privilege have potential to be therapeutically adapted to suppress inflammation within the eye and in other tissues.

One of the experimental examples of ocular immune privilege is the phenomena of anterior chamber associated immune deviation (ACAID) (13, 14). ACAID is induced by placing foreign antigen within the anterior chamber of the eye. The antigen is picked up and processed for presentation by F4/80 positive macrophages that migrate to the spleen (15, 16). In the spleen with the help of recruited B-cells and NK T cells, there is an antigen-specific activation and expansion of both CD8 and CD4 regulatory T cells (17–20). This brings about systemic tolerance to the foreign antigen. Placing foreign antigen in the subretinal space (the temporary pocket that forms when the photoreceptors are detached from the RPE) also induces an ACAID-like response (21). This has defined immune privilege to include the retina.

### RPE Regulation of Immune Activity

The placement of neonatal-retinal allografts into the retina are not immunologically rejected and moreover they differentiate (22). Also, there is induced tolerance to the alloantigen through an ACAID-like response. The induction of the ACAID-like response is mediated by TGF-β2 like in ACAID; however, it requires the expression of Thrombospondin-1 (TSP-1) (23). The TSP-1 is a known activator of latent TGF-β2 (24). In mice with TSP-1 knocked out the ACAID-like response cannot be induced; moreover, TSP-1 knock-out mice with experimental autoimmune disease (EAU) cannot self-resolve EAU like wild-type mice. The ACAID-like response cannot be induced when the integrity of the RPE monolayer is compromised through chemical or laser wounding (21, 25, 26). In addition, laser wounding not only causes the loss of immune privilege in the affected eye but also causes a loss of immune privilege in the untouched contralateral eye. This may be mediated by the release of Substance P by the retina (26). Together the results demonstrate the need for an intact RPE monolayer to maintain ocular immune privilege.

The ACAID-like response shows that the RPE directly affect the functionality of immune cells by maintaining the anti-inflammatory retinal microenvironment. One of the interesting findings is that the RPE soluble molecules induce and enhance regulatory activity in Treg cells (27–29). Also, the RPE release soluble factors that suppress the activation of effector T cells (23, 30). Of interest in this review is the ability of the RPE to regulate potential antigen presenting cells, the immune cells that sit at the interface of innate and adaptive immune response. The RPE have been found to suppress the activation of dendritic cells (31, 32), which would prevent naive T cell activation to antigen carried from the retina to a regional lymph node. In addition, the RPE promote the development and activation of myeloid suppressor cells from bone-marrow progenitor cells (33). These suppressor cells are highly capable of preventing and inhibiting adaptive immune responses. Interestingly, the RPE induction of these suppressor cells is mediated by IL-6, which is usually considered a proinflammatory cytokine and an anti-ACAID cytokine (34).

Using a technique of *in situ* RPE eyecup cultures, treating endotoxin-stimulated macrophages with the RPE eyecup conditioned culture-media suppresses proinflammatory cytokine production, while promoting anti-inflammatory activity (35–37). Moreover, the treatment of macrophages with the RPE soluble factors induces anti-inflammatory cytokine production and characteristics of myeloid suppressor cells (37). The mediators of this activity are the neuropeptides α-MSH, and Neuropeptide Y (NPY) produced by the RPE. The collective action of the soluble factors, constitutively produced by the RPE, induce macrophages, and resident microglial cells to be themselves mediators of anti-inflammatory activity and activators of Treg cells. These findings demonstrate the importance of the RPE monolayer in maintaining immune privilege.

## RPE Physiology

### RPE Function

The health and integrity of the RPE monolayer may very well be required for maintaining immune regulation along with maintaining a functional retina. The RPE is a cuboidal monolayer of hexagonal cells that lies between the photoreceptors and Bruch’s membrane (38). On the apical side, the RPE has microvilli that envelop the distal-ends of the photoreceptors with each RPE cell projecting towards 20-55 photoreceptors (39–41). On the basal side lies Bruch’s membrane a pentalaminar structure, which separates the RPE from the eye’s fenestrated choroidal capillaries (42). The RPE maintains this polarity through a complex network of tight junctions near the apical side that create a barrier to paracellular diffusion (43). The tight junctions include occludins and claudins both of which play essential regulatory roles in maintenance of the function of the tight junctions. The composition of claudins expressed in the RPE varies by species (44). In the human RPE, claudin 19 is the most predominant and mutations of CLDN10 gene that encodes it can lead to dysfunction of the tight junctions along with severe ocular abnormalities (43, 45).

The RPE plays a variety of critical roles in maintaining the function of the retina including acting as the outer blood-retina barrier and regulating the transport of waste and nutrients (46, 47). In order to accomplish these functions, the RPE exhibits polarity with an asymmetric distribution of organelles, proteins, and functionality allowing it to create a unique microenvironment for the retina (48). For example, melanosomes are preferentially located near the apical cell membrane with Golgi and mitochondria preferentially localize to basal cytoplasm (38). The visual cycle is dependent on the conversion of 11-cis-retinol to 11-trans-retinol and the RPE plays a key role in re-isomerizing 11-cis-retinol from 11-trans-retinol (39, 49, 50). Many of the key metabolic enzymes involved in this re-isomerization process are expressed in the RPE (39). The photoreceptors have a delicate equilibrium between nutrient renewal and damaged component disposal which sheds up to 10% of their volume. The RPE phagocytizes the end-processes allowing new end-processes to take their place (39).

### RPE and the Retinal Blood Barrier

In the healthy retina, the RPE separates the choroidal blood supply from photoreceptors and manages the microenvironment of the retina by regulating the flow of water and ions between the two spaces (51). The RPE contains tight junctions that play a role in its ability to act as the outer part of the blood-retina barrier (47). In studies on chicken RPE, it was shown that the tight junctions of the RPE have increased complexities with P-face-associated tight junctions *vs* the tight junctions in choroid vessels (52). This increased complexity may be necessary for the formation of an effective blood-retina barrier. However, *in vitro* studies have indicated that the functional barrier for macromolecules, specifically serum albumin, is similar between the RPE and the iris pigment epithelium (53). This suggests that at the very least the general barrier function of the RPE is like other tight-junction epithelial layers. The regulation of what can enter the retina is apparent as the outer blood-retinal barrier formed by the RPE sometimes poses a problem in the use of some systemic drugs for the treatment of retinal diseases (54). However, even with the blood-retina barrier of the RPE and its tight junctions, the retina is still susceptible to damage, as in the case of some systemic medications lead to retinal dysfunction and degeneration (21, 55).

In disease states like AMD and Alzheimer’s, the accumulation of Aβ in the RPE *via* RAGE/p38 MAPK-mediated endocytosis can lead to attenuation and disorganization of the tight junctions, and in some cases breakdown of the tight junctions (56, 57). In AMD the leading cause of visual impairment in western countries of people over 50 (58), damage to the RPE and RPE dysfunction are thought to be the initial insult in the atrophic variant which accounts for 85-90% of cases (40, 59). In the wet variant, while the choriocapillaris complex is thought to be the initial site of dysfunction, damage to the RPE soon follows and plays a key role in visual loss (59).

The breakdown of the blood-retina barrier created by the RPE has been shown to be one of the earliest pathologic changes that can be detected in some diseases like diabetes (60). In diabetic retinopathy, previous work has noted that the involvement of the inner blood-retina barrier due to endothelial cell dysfunction can lead to diabetic macular oedema and retinopathy (47). The dysfunction of the outer blood-retina barrier at the RPE may play a role in diabetic retinopathy in that the presence of cytokines, such as IL-6, have been shown to disrupt the outer blood-retina barrier through amplified recruitment of microglial cells and increased production of TNF- α (61). Additionally, high glucose states have been shown to lead to RPE cells downregulating GLUT-1 and a reduction in the levels of antioxidants potentially leading to retinal tissue damage (62). In some rodent studies the breakdown of the tight junctions in the RPE results in vascular leakage as visualized in diabetic and ischemic rodents (63). When the blood-retina barrier is compromised, it can lead to additional disease processes such as uveitic macular edema (64). Abnormal regulation of the RPE in mice lacking ATP-binding cassette transporters (ABCA1 and ABCG1) leads to discontinuities of the RPE and degeneration of the overlying photoreceptors (65). In some disease states like AMD and diabetic retinopathy the transplantation of RPE has even been explored as treatment options with some trials showing preliminary signs of success (66, 67). Interestingly in mice with degenerating photoreceptors, retinal microglia cells migrate from the inner retina to the subretinal space and undergo a transcriptional change to express homeostatic checkpoint and wound-responsive genes that protect the RPE (68). While it is not clear as to the signals that induced the migration, this does suggest that there is an initial attempt by the ocular microenvironment to preserve the functionality of the RPE and its blood-barrier under disease conditions. Therapeutic interventions that can maintain or restore RPE health could also maintain and restore immune privilege that would reduce the potential contribution of inflammation to many retinal degenerative diseases.

## Changes in the RPE Experimentally Induced Disease

### Laser Injury

The growing use of lasers in the military, healthcare, laboratories, and academia causes an increased in ocular injury by misdirected lasers (69). In general, lasers are classified as I, II, IIIa, IIIb and IV. The first two Classes of Class II and Class IIIa are relatively safe while the last two are hazardous. Laser light is visible between 400 and 700 nm, and other sources are infrared and ultraviolet lasers. Usually, transient exposure to Class II or Class IIIa laser may not result in eye injury. Handheld laser pointers are usually Class IIIa and have comparatively low energy that is insufficient to cause injury at the ocular surface, but the focusing power of the eye causes a powerful amplification of irradiance that makes the retina susceptible to laser injury. The prolonged exposure to this laser may cause severe, permanent, and irreversible damage accompanied with vision loss (70, 71).

**Figure 1:**
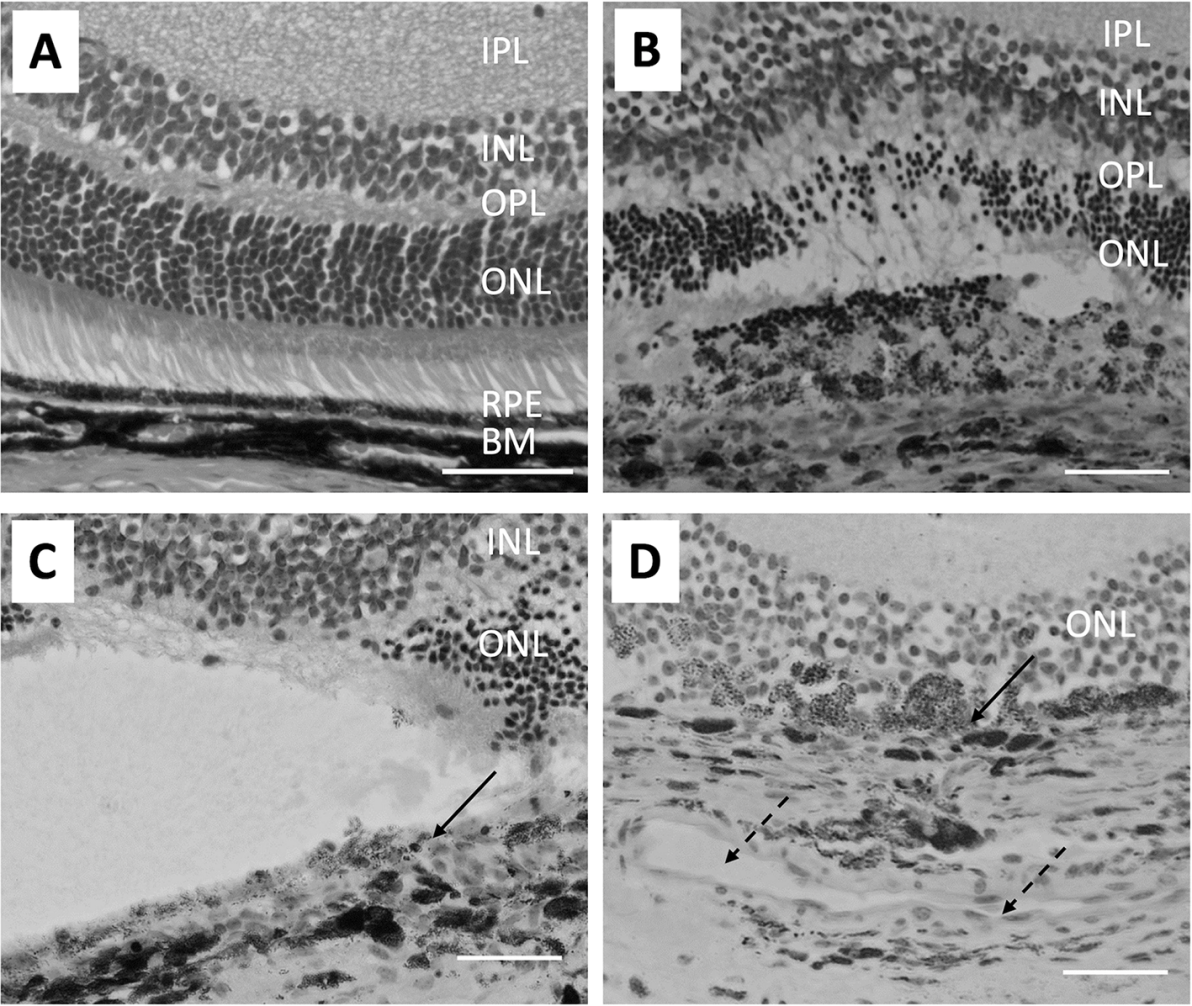
Micrographs show the pathological changes of retina and choroid from day 0 to day 14 after laser injury. **(A)** shows a healthy naive retina. The RPE is a single cell layer. **(B)** shows the retina on day 3 after laser injury. Bruch’s membrane is disrupted and RPE cells and most ONL cells are lost at the laser injury site. Also, arrangement of pigmented cells in the choroid is disrupted. **(C)** Similar histological structures are seen on day 5 after retinal laser-injury. Solid arrow points to the discontinued RPE cells. **(D)** shows the retina 14 days after laser injury. A large capillary (broken arrows) forms in the choroid adjacent to the laser injury site. Macrophages filled with pigment are found in the ONL and some RPE (solid arrow) are found covering the injured site with a disrupted Bruch’s membrane. The size bar is 50 microns in length.

The coagulative irradiation of laser causes an initial destruction and secondary tissue responses (**Figure 1**). The initial destruction occurs from the thermal degradation of the incident energy absorbed by RPE pigment and changes are seen almost immediately (72–75). Coagulation necrosis of the RPE and the photoreceptor cells happens at this acute stage. The inner-nuclear-layer (INL) is usually unharmed. The RPE barrier breaks down and cell debris is found within and between disrupted RPE and in the outer retina. Three days after laser burn, Bruch membrane is disrupted and the subretinal space and the inner retina are infiltrated with macrophages. The RPE and photoreceptors become necrotic (**Figure 1B**). Five days after laser burn, all photoreceptor cells around the burn area are gone, but most of the INL cells are still intact (**Figure 1C**). There is vacuoling of cells in the IP and the retinal ganglion cell (RGC) layers are seen. By day 14, the RPE show placoid and are found to cover the laser injury site partially. There were many clusters of pigment-filled macrophages around the injury site including in the INL (**Figure 1D**). By day 120, the RPE covers the injury site (75).

The RPE is not only a physical barrier but is also an important source of immune-suppressive molecules that contribute to the immune privileged status of the eye. In naive retinas, microglia are in the inner and outer plexiform layer and after laser injury they accumulate with macrophages and granulocytes at the site of the laser burn in the retina and choroid (76). Changes in retinal microglial cells can be seen 1 day after the laser injury with expression of MHC class II (75). In addition, co-stimulatory factors of CD40 and CD86 are found on these activated microglia. The condition media of cultured RPE eyecups from laser-wounded eyes contain significantly lower amounts of α-MSH (37). In the normal retina the microglia co-express NOS2 and Arginase1, but in laser wounded retinas co-expression of the two enzymes is not seen (37). Moreover, infiltrating the laser wound site are Arginase1-positive macrophages that are a source of VEGF to initiate choroidal neovascularization (37, 77). This corresponds with the loss of ACAID in both the eye with the laser injury and the unwounded counter-lateral eye (25, 26). This appears be mediated by the release of another neuropeptide Substance P. How this affects RPE regulation of immunity in the non-lasered eye is not understood; however, the laser wounded RPE monolayer does not induce the co-expression of NOS2 and Arginase 1 in macrophages (37). This further indicates the importance of an intact RPE monolayer for the RPE to regulate immune cells.

After the laser injury, the damage of the RPE and the surrounding neural retina and the underlying choroid, the retinal microglia and the choroidal inflammatory cells may mediate release of proinflammatory cytokines (78–80). These cytokines may exacerbate neuronal damage. Pro-angiogenic VEGF is the primary factor made and pro-inflammatory cytokines including IL-1β, IL-3, IL-6 and TNF-α would be necessary as a wound repair process following laser photocoagulation. In addition, production of chemokines including MCP-1 and MIP-2 are increased (75, 81). These show that physical damage of the RPE monolayer promotes the infiltration of immune cells and the induction of an inflammatory response. This has implications not only on laser wounding, but also on retinal degenerative diseases like age related macular degeneration as RPE cells die.

### Experimental Autoimmune Uveitis

Autoimmune uveitis is one of the leading causes of blindness in developed countries. The retina is usually the target, but the RPE cells may also be killed as collateral damage due to inflammation (82, 83). The common rodent models of experimental autoimmune uveitis (EAU) are induced with inter-photoreceptor retinoid-binding protein (IRBP) emulsified in adjuvant, and other models use retinal arrestin, rhodopsin and RPE-65 (84–86). The IRBP-model has a prodromal phase till day 14 and usually reaches a score of 1 on the clinical grading system and peaks on day 21 with a clinical score of 3. Then the disease progresses into a chronic phase of sustained clinical scores of 3 until day 70 where the disease begins to self-resolve (87, 88).

**Figure 2:**
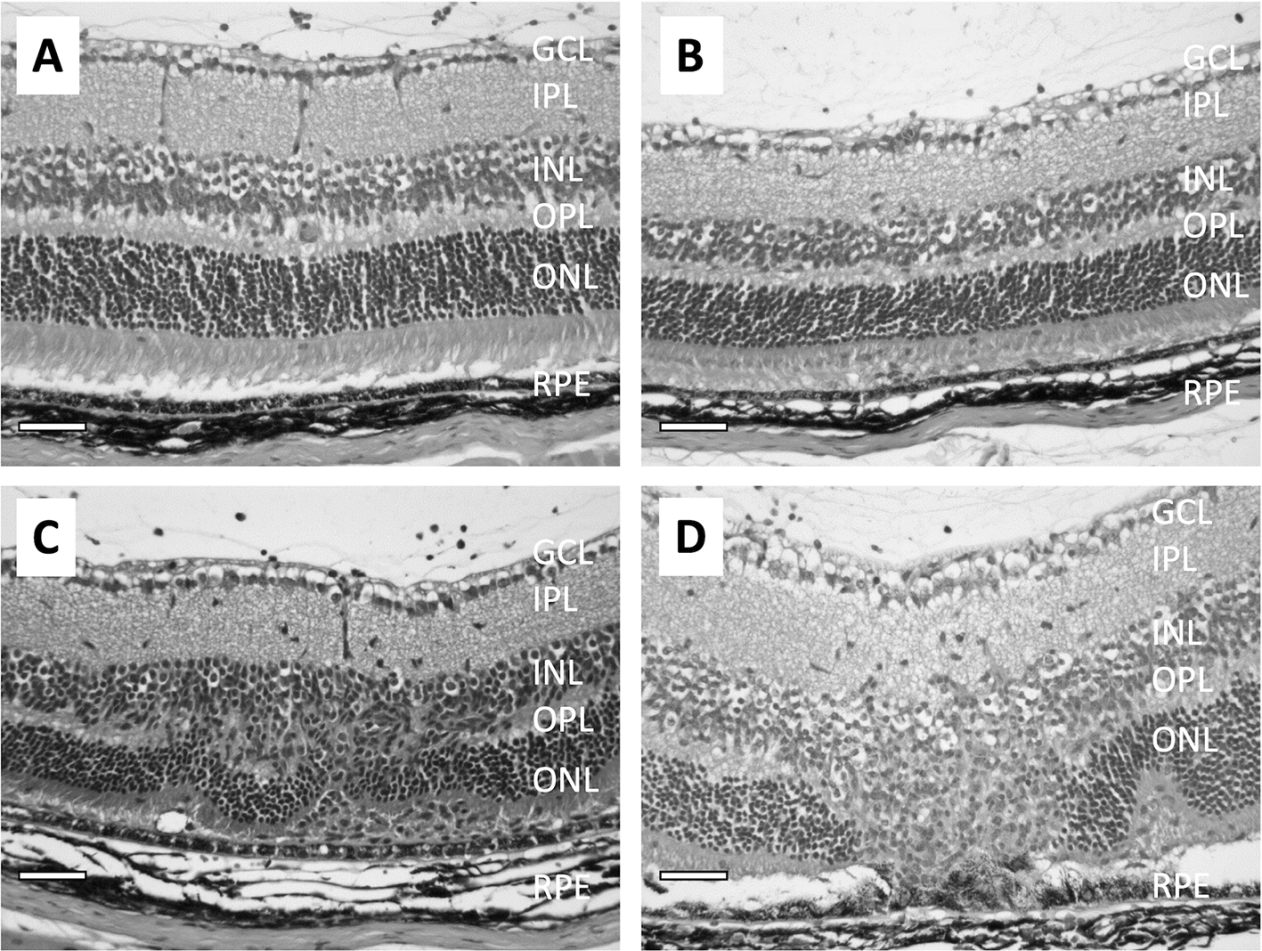
Micrographs show the pathological changes of retina from mice with EAU clinical score ranging from 1 to 3. **(A)** shows a retina with EAU clinical score 1. The RPE is intact and there are no noted changes in the retina except for some inflammatory cells in the vitreous. **(B)** shows the retina with EAU clinical score 2. There are more infiltrating cells in the vitreous and the subretinal space. Both the retina and RPE are intact, with minor lesions found in the INL. **(C)** is the retina at early stage of chronic EAU, clinical score 3. The RPE is intact, and infiltration of inflammatory cells are found in the vitreous and in the retina with disruption of the INL and IPL with loss of the ONL cells. **(D)** shows the retina at a late stage of chronic EAU, sustained clinical score of 3. The RPE monolayer is disrupted and fused with the ONL and adjacent outer/inner segments of the photoreceptors gone. There are pigment-filled macrophages around sites of RPE cell loss. The size bar is 50 microns in length.

Histological evaluation of the eyes from rodents immunized to induce EAU show that the loss of the RPE monolayer is progressive along with the chronic nature of EAU (**Figure 2**) (89–91). Very little damage to the RPE is seen in the early stages of EAU (**Figures 2A–C**); however, As the disease progresses through the chronic phase, there is severe damage of both INL cells and photoreceptor cells, with the RPE shows damage with pigment-laden macrophages near the damaged RPE (**Figure 2D**).

The pathogenesis of inflammatory disorders of EAU is associated with autoreactive effector CD4+ T cells. In the early stages of IRBP-induced EAU the effector T cells are polarized to the Th1 phenotype and produce IFNγ (92, 93). These effector Th1 cells are highly active and mediate EAU in naive recipient animals. However, neutralizing IFNγ does not suppress EAU but worsen the disease because of the activation of Th17 cells (94–97). The Th17 cells target the RPE, and the disruption of the blood-retinal barrier causes the influx of serum antibodies, which exacerbate EAU (98). Mice with TSP-1 knocked-out also suffer with severe and prolonged EAU. To initiate a T cell response there needs to be present an APC expressing MHC class II. Normally the expression of MHC along with co-stimulatory molecules within the eye is low to undetectable, and while initially the microglia do not express MHC, they increase in MHC expression as EAU progresses (93, 99). In addition, there is need for the microglia in a non-MHC dependent manner to recruit effector T cells and MHC-expressing monocytes into the retina (99).

Since the course of EAU is self-limiting, it has suggested that while the RPE may be targeted and affected by the retinal inflammation there is still some immunosuppressive activity. It was found that EAU resolution is associated with the emergence of a specific type of APC within the spleen that in an antigen-specific manner counter-converts effector T cells into inducible Treg cells (88, 100). This process is dependent on the expression of the melanocortin 5-receptor (MC5r), one of the receptors of α-MSH. Moreover, expression of MC5r is necessary to modulate the severity of EAU and the functions of APC (91). The RPE is important to maintain the health of the retina not only by phagocytosis of photoreceptor outer segments, recycling retinol and maintaining the blood-retinal barrier, it also provides support to maintain immune privilege within the eye.

## RPE and Regulation of Immunity

### RPE Influenced Activity of Macrophages

ACAID demonstrates that macrophages with the potential of becoming antigen presenting cells (APC) are influenced by the ocular microenvironment to promote Treg cell activation. This has suggested that within the retina a similar influence should be seen with resident microglial cells. When assayed it is found that microglial cells are very much suppressed in immune activity; moreover, they co-express Nitric Oxide Synthase 2 (NOS2) with Arginase 1 (37). This co-expression of a M1 marker of inflammation-mediating cells and a M2 tissue repair/suppressor-mediating cells is characteristic of tumor associated myeloid cells that suppress immune attack of tumors (101, 102). Co-expression of NOS2 and Arginase 1 is induced by treating macrophages with the soluble factors of RPE. Specifically, it was found that the neuropeptides α-MSH and NPY produced by the RPE mediate this unique characterization of macrophages, which can enhance apoptosis in activated effector T cells (37). The soluble RPE factors also induce macrophages to produce anti-inflammatory cytokines even when the macrophages are treated with a pro-inflammatory signal such as endotoxin (35, 36, 103). This alternative activation of macrophages is mediated by α-MSH and provides for an anti-inflammatory response when there is an immune challenge within the ocular microenvironment (35). This potential for the retina to be a site of generating alternatively activated macrophages and myeloid-like suppressor cells makes the environment nor only anti-inflammatory but a site where immune cells are made to regulate other immune cells.

Phagocytizing materials is central for an APC to process antigen for presentation on MHC class II molecules (104). Recently we have found that the process of phagocytosis is also altered by the RPE through its release of α-MSH and NPY (105, 106). The neuropeptides mediate a differential regulation of phagocytic uptake of gram-negative and gram-positive bacteria (107). There is suppression in the number of gram-negative bacteria taken up and a suppression of the number of macrophages taking-up gram-positive bacteria. There is no change in the expression of scavenger receptors on the macrophages suggesting that this may be a change in how Toll-like receptor stimulation in the macrophages is suppressed (108, 109). If the bacteria are opsonized, there is no effect of the neuropeptide treatment on the up-take of opsonized-gram-negative and positive bacteria (107). However, activation of the phagolysosome is suppressed. The suppression is in part due to both downregulation of LAMP1, which is needed for phagolysosome acidification, and a blockade of the phagosome maturation pathway preventing the transition of phagosomes from early to intermediate (110). The suppression of phagocytosis and phagosome maturation by the RPE through the neuropeptides α-MSH and NPY would either prevent the processing of antigen within the retina or at least alter the processing of the antigen to unrecognizable amino-acid sequences that could be presented by APC in the retina (110). The RPE from eyes with autoimmune uveitis do not suppress the phagocytic pathway, and this may be associated with high levels of IL-6 expression (105). The regulation of phagolysosome activation is dependent also on the RPE maintaining an intact monolayer (106). Therefore, one potential contribution of the RPE to immune privilege is its suppression of the processing and presentation of self-antigens by retinal APC that would prevent the activation of autoimmune disease-mediating effector T cells. This importance of the RPE to mediate immune regulation and prevent induction of autoimmune disease indicates that changes to the RPE will have a corresponding change in the regulation of immune cells within the retina.

### Recovery of RPE Mediated Immune Regulation

A key reason for understanding the molecular mechanisms of ocular immune privilege is to see whether these molecules that are normally regulating immune cell activity can be used to suppress uveitis and restore immune privilege. Since the neuropeptide α-MSH has its own immune regulating/anti-inflammatory properties as well as contributing to the mechanism of ocular immune privilege there is a strong potential of using α-MSH as a therapeutic approach to uveitis (111, 112). The neuropeptide is 13 amino acids long and is easily injected. When mice with EAU are treated with α-MSH at the beginning of the chronic phase the retinal inflammation begins to resolve within a week of the treatment (84, 91, 100). After 2 to 3 weeks EAU is fully resolved in comparison to another 8 weeks in the untreated EAU mice. In the spleen of the α-MSH-treated EAU mice are Treg cells specific for retinal autoantigen. These Treg cells provide the mice with protection from a memory immune response to the autoantigen. The same induction of Treg cell is found in the spleen of mice that have resolved EAU on their own (88, 113, 114). These Treg cells are derived from the population of effector T cells that are converted by APC into antigen-specific inducible Treg cells (88, 100). Under conditions of uveitis the RPE cannot suppress phagosome maturation and phagolysosome activation, and after α-MSH therapy the RPE recover their ability to suppress phagosome maturation and phagolysosome activation (91, 105). Therapeutic use of α-MSH in EAU suppresses uveitis, induces Treg cells to retinal autoantigen, and may very well reestablish RPE regulation of immune cell activity within the retina.

There is a dependency on MC5r for some of the EAU recovery. While MC5r-knockout mice recover on their own from EAU, like wild type mice, they lack the presence of the suppressor APC and the antigen-specific Treg cells within their spleens (100, 114, 115). While α-MSH treatment suppressed EAU in the knockout mice it does not protect the retina from the damage of inflammation, not did it promote recovery of RPE mediated suppression of phagolysosome activation (91). These results suggest that while the use of α-MSH in therapy to suppress inflammation is possible through its other receptors, it appears that the recovery of immune privilege is dependent on α-MSH working through MC5r. Also, by tailoring the therapy to specific melanocortin receptors different aspects of an immune response can be targeted for regulation (116). The experimental therapeutic use of α-MSH in EAU demonstrates that it is possible to use the mechanisms of ocular immune privilege, and potentially other RPE generated molecules, to suppress inflammation and reestablish ocular immune privilege and tolerance (117, 118).

## Conclusion

The RPE holds an important role in the maintenance of ocular immune privilege in being a blood-barrier and a producer of immune regulatory molecules. These molecules are not just anti-inflammatory, they promote immune regulatory and suppressive behaviors within immune cells they target. It makes these different for other forms of therapy that either suppress all immune activity or block specific key cytokines. The change in immune cells behavior by the RPE allows for immune cell activity to be supportive of the retina while immune cells regulate themselves and other immune cells that may migrate into the retina. While it is not fully understood as to whether in retinal diseases the change is first in the retina or in the RPE, but once the RPE layer is injured it is difficult for the retina to function and to prevent the activation of damaging immune activity.

## Author Contributions

All three authors were involved in the planning, writing and editing of this review and agree to be accountable for the content of this work. All authors contributed to the article and approved the submitted version.

## Funding

This work is supported in part by a grant from the NIH/NEI EY025961 and the Massachusetts Lions Eye Research Fund.

## Conflict of Interest

AT is a scientific advisor and recipient of a sponsored research agreement from Palatin Technologies Inc.

The remaining authors declare that the research was conducted in the absence of any commercial or financial relationships that could be construed as a potential conflict of interest.

## Publisher’s Note

All claims expressed in this article are solely those of the authors and do not necessarily represent those of their affiliated organizations, or those of the publisher, the editors and the reviewers. Any product that may be evaluated in this article, or claim that may be made by its manufacturer, is not guaranteed or endorsed by the publisher.
